# Exogenous melatonin delays strawberry fruit ripening by suppressing endogenous ABA signaling

**DOI:** 10.1038/s41598-023-41311-1

**Published:** 2023-08-30

**Authors:** Sirvan Mansouri, Mahmoud Koushesh Saba, Hassan Sarikhani

**Affiliations:** 1 Department of Horticultural Science, Faculty of Agriculture, University of Kurdistan, Sanandaj, Iran; 2 Research Center of Strawberry Improvement and Breeding, University of Kurdistan, Sanandaj, Iran; 3 Department of Horticultural Science, Bu-Ali Sina University, Hamedan, Iran

**Keywords:** Hormones, Biotechnology

## Abstract

Ripening as a physico-chemical change is part of a continuous developmental process and hormones play a major role in this processes. The present study was carried out to investigate the effect of external melatonin (0 and 10 μM) injection at the light green stage on the ripening of strawberry fruit. The fruit was sampled for morphological, biochemical, and gene expression analysis during (0, 5, 10, and 15 days after treatment). The results showed a lower accumulation of anthocyanin content was observed in fruits treated with 10 μM. The injection of 10 μM melatonin caused a lower total soluble solid content and fruit color, and higher titratable acidity and softening. The total phenol content was higher in fruit treated with 10 µM melatonin, accompanied by increased PAL enzyme activity and gene expression, increased DPPH scavenging capacity, and higher content of quercetin, gallic, caffeic, and chlorogenic acids. The delay in fruit ripening was associated with suppression of H_2_O_2_ level and endogenous ABA accumulation caused by lower expression of *NCEDs* genes. In general, it is concluded that activating the melatonin ROS scavenging cascade might be responsible for the delayed ripening and development of strawberry fruit. Therefore, our study demonstrates that the exogenous application of 10 μM melatonin can slow the ripening of strawberry fruit.

## Introduction

Fruit ripening is a coordinated process influenced by genetic, physiological, and biochemical factors. These processes lead to changes in color, texture, aroma, taste, odor, and nutritional quality^1^. The mechanism that regulates ripening is not fully understood in non-climacteric fruit but may be related to changes in hormonal concentrations^2^ . Studies have shown that ABA plays a crucial role in the ripening of non-climacteric fruits while, in climacteric fruits, the role of ethylene in the ripening is widely known^3^. Recent studies showed ABA and melatonin have positive or negative interaction and influenced several physiological process including stress tolerance and fruit ripening^4–6^.

Melatonin (*N*-acetyl-5-methoxytryptamine) is an indoleamine synthesized through tryptophan metabolism from serotonin^5^. Melatonin, as an antioxidant and signal molecule, prevents adverse effects of stress in plants by (1) increasing plant resistance to biological and non-biological stresses such as salinity, drought, cold, and microorganisms^7^ and by (2) inhibiting ROS activity^8^. Due to its hydrophilic and lipophilic structure, melatonin quickly penetrates cells and has higher antioxidant activity than some vitamins^9^ . Furthermore, Wei et al.^10^ reported that melatonin, as a phytohormone, plays a receptor-dependent signal role, and its uptake through G protein-coupled (PMTR1 or CAND2) receptors in plasma membranes are essential for closing pores by controlling H_2_O_2_ and Ca^2+^ signaling.

The role of exogenous melatonin in delaying banana fruit ripening is through reduced starch degradation and decreasing the expression of genes involved in ethylene biosynthesis (*ACO* and *ACS*)^11^. Tijero et al.^5^ reported that 100 µM melatonin treatment delayed the ripening of the unripe cherry fruit by increasing internal melatonin and internal cytokinin and preventing anthocyanin accumulation in the fruit. Liu et al.^12^ also suggested that treating pear fruit with 100 µM melatonin increased fruit firmness after harvest by reducing the expression of *ACO* and *ACS* genes, reduced ethylene biosynthesis, and prevented *PG* expression and *Cel* genes. In contrast with above studies, the effect of melatonin in non-climacteric fruit has shown that 1000 µM melatonin treatment accelerates the ripening of strawberry fruit by increasing anthocyanins and endogenous ABA^4^. Xu et al.^6^ reported that melatonin treatment (100 μM) promotes grape berry ripening by increasing anthocyanin levels than control samples. They found that ripening accelerated by increasing endogenous anthocyanin content. Similar to conflict effect of melatonin on ripening, methyl jasmonate also had different effect on ripening depending to its concentration^13^. Therefore, more study is needed to reveal melatonin impact on non-climacteric fruits.

Strawberry (*Fragaria* × *ananassa* Duch.) is a globally popular fruit with an attractive appearance and a rich source of anthocyanins, flavonoids, phenolic compounds, and folate. Strawberries, which are non-climacteric, should be harvested at full maturity to achieve maximum edibility^14^. This fruit is also highly perishable due to low mechanical resistance, high respiration rate, and high susceptibility to pathogen attack. Some undesirable changes observed during postharvest include weight loss, accelerated softening, mechanical injury, and *Botrytis cinerea*-induced decay^15^. Aghdam and Fard^16^ reported that the reduction of decay in strawberry fruit treated with melatonin during storage might be due to the accumulation of H_2_O_2_ signaling to stimulate the GABA shunt pathway, providing sufficient intracellular ATP along with the accumulation of more phenols and anthocyanins and as a result increasing the DPPH inhibition capacity. Liu et al. (2018) reported that the reduction of decay of strawberry fruit treated with melatonin during storage may be due to endogenous melatonin accumulation. In addition, Pang et al.^17^ reported that strawberry fruit treated with melatonin showed better overall quality, represented by lower weight loss, higher firmness, higher DPPH scavenging capacity, higher phenols, anthocyanins and ascorbic acid accumulation.

Adequate knowledge about physiological, biochemical, and molecular regulatory mechanisms associated with fruit ripening will help improve strategies to delay ripening and enhance marketability. This experiment aimed to study the effect of melatonin on strawberry fruit ripening and the expression of genes involved in the regulation of development and ripening. In addition, the effects of exogenous melatonin treatment on melatonin biosynthesis-related genes and antioxidant systems were evaluated.

## Materials and methods

### Plant materials and melatonin treatment

Strawberry (*Fragaria* × *ananassa* cv. Sabrina) plants were purchased from a commercial unit affiliated with the Agricultural Research Center and Natural Resources of Kurdistan province, then were grown in a greenhouse under controlled conditions (20–25 °C, relative humidity 70–85%) in University of Kurdistan. Melatonin concentrations (10 μM) and distilled water (used as a control) were injected into the fruit: 200 μL of the treatment solutions were injected by sterile hypodermic syringe into the fruit core through the pedicel, at 10 days after flowering (DAF) that fruit are in light green (the fruit begins to expand rapidly and becomes much larger) stage^4, 18^. Thirty uniformly sized fruit per replicate were sampled at 0 (before treatment), 5, 10, and 15 days after treatment. Fifteen fruit per replicate of each treatment were chosen to evaluate color, firmness, total soluble solid (TSS), and titratable acid (TA). For biochemical analysis and gene expression, fifteen more fruit in each replicate of each treatment were frozen in liquid nitrogen, powdered, and then quickly stored at − 80 °C.

### Endogenous anthocyanin content

Anthocyanin extraction was carried out according to Bodelon et al.^19^. Briefly, 2 g frozen fruit powder was homogenized with 18 mL 0.5% (v/v) HCl in methanol at 4 °C for 1 h to extract the pigments. Then filtered by a single layer of Whatman No 1. The absorbance of the resulting clear liquid was measured at 520 nm (maximum absorbance for anthocyanins). Anthocyanin content was calculated using the following formula: Abs520 × dilution factor × (molecular weight of pelargonidin × molar extinction coefficient) and expressed as g pelargonidin equivalent (PE)/kg fresh weight (FW).

### Fruit firmness, TSS, TA, and color

Fruit firmness was measured using a penetrometer with an 8-mm tip diameter (FHT200, Chine) and expressed in Newton (N). A refractometer (Atago, Tokyo, Japan) was used to determine the TSS of fruit juice at 20 °C. TA was determined by diluting the remaining juice (1/10, v/v) and titrating with NaOH to pH 8.2, and expressed as TA % citric acid. Using a colorimeter (TES 135 A, Taiwan), the color was measured on two opposite sides (equatorial region), and a* parameter was recorded to represent the redness. The color was measured at 10 and 15 days after treatments when fruit color changed.

### Endogenous melatonin accumulation

Melatonin extraction was done at 4 °C, according to Arnao and Hernandez^20^. Briefly, 2 g frozen fruit powder was homogenized in vials containing 3 mL chloroform. The samples were shacked at 4 °C in darkness for 15 h and then filtered by Whatman No. 1. The extract were evaporated to dryness in freeze dryer (RV06-ML, Germany), and the residue was dissolved in acetonitrile (0.5 mL). The endogenous accumulation of melatonin was analyzed with HPLC equipment (Knuer Scientific Instruments, Berlin, Germany) using a C18 column (250 mm × 4.6 mm length, 5 μm particle size) with a fluorescence detector, and the results were expressed as μg/kg FW. The isocratic mobile phase consisted of water: acetonitrile (50:50) at a flow rate of 0.2 mL/min.

### Endogenous ABA accumulation

Frozen fruit powder (2 g) was homogenized with 70% (v/v) methanol and stirred overnight at 4 °C according to the methods of Kelen et al.^21^. The extract was filtered by Whatman filter (No.1), and the methanol evaporated under vacuum. Residue was dissolved in 0.1 M phosphate buffer, and same amount of ethyl acetate was added to the obtained solution to dissolve impurities. After removing the ethyl acetate phase, by 1 N HCl, the pH of the aqueous phase was adjusted to 2.5. The solution partitioned by diethyl ether and then passed through anhydrous sodium sulfate. Later, under vacuum, the diethyl ether phase was evaporated. Then, the dry residue containing ABA was dissolved in 2.0 mL of methanol, and used for ABA analysis. The ABA was analyzed with HPLC equipment (Knuer Scientifc Instruments, Berlin, Germany) using a C18 column (250 mm × 4.6 mm length, 5 μm particle size) at a 0.8 mL/min flow rate. An injection volume of 20 µL was used for each analysis. The endogenous content of ABA was monitored at 265 nm and expressed as mg/kg FW.

### Endogenous H_2_O_2_ accumulation

Endogenous H_2_O_2_ accumulation was expressed as the reaction of H_2_O_2_ with potassium iodide as Alexieva et al.^22^ stated. Briefly, frozen fruit powder (1 g) was homogenized in a mortar containing 10 mL of 0.1% (w/v) trichloroacetic acid. The homogenate was centrifuged at 12,000 g for 15 min. Then, 500 μL of the supernatant solution was added to 500 μL of potassium phosphate buffer 10 mM (pH = 7) and 2 mL of potassium iodide. The resulting mixture was placed in the dark for 1 h at 25 °C. The absorbance of the samples was measured at 390 nm using spectrophotometer (Carry 100, Varian, USA). The endogenous H_2_O_2_ accumulation was expressed as mmol/kg FW.

### Activity of PAL enzyme

The PAL enzyme extracted according to Yao et al.^23^. Frozen fruit powder (0.5 g) was ground with 5 mL borate buffer (pH 8.8) contained 15 mM β-mercaptoethanol and 0.15% w/v PVP. The homogenate was centrifuged at 10,000 g for 20 min at 4* °C*. For assaying PAL activity, the supernatant was used as a source of crude enzyme. The reaction mixture contained 0.5 mL of the supernatant, 1 mL of 0.02 mol/L l-phenylalanine and borate buffer (0.05 mol/L, pH 8.7). Incubation was performed at 30* °*C for 1 h, and the reaction was stopped by adding 1 mL of 2 M HCl. The absorbance was measured at 290 nm. The activity of the PAL enzyme was expressed as mmol cinnamic acid/h kg FW.

### Endogenous accumulation of total phenols (TP) and DPPH scavenging capacity

Based on the method of Dewanto et al.^24^ and using the Folin-Ciocalteu reagent, TPC content was measured. The absorbance was calculated and measured as g gallic acid equivalent (GAE) (PE)/kg FW. DPPH scavenging capacity was measured using a spectrophotometer (Carry 100, Varian, USA) based on the method represented by Brand-Williams et al.^25^. The following equation was used to calculate the extracts' DPPH free radical scavenging activity. DPPH scavenging effects (%) = [(A0−A1*/*A0) × 100], where A0 absorbance in the absence of extract, A1 absorbance in the presence of solution.

### RNA extraction, synthesis of cDNA and quantitative real-time PCR

The RNA was extracted by a Kit (Takara, Japan) according to the manufacturer’s protocol and the list of the gene-specific primers’ sequences was shown in Table 1. RT-qPCR was conducted using SYBR Premix Ex Taq (Takara, Japan) conforming to the producer’s manual. RNA was reverse-transcribed into cDNA using a PrimeScript RT reagent kit (Takara, Japan) conforming to the producer’s manual. The final volume containing 1 μL of cDNA, 100 nM primers, and 5 μL of 2 × SYBR GREEN I Master Mix (Takara, Japan) was prepared conforming to the producer’s manual. The comparative threshold cycle (Ct) value method calculated the relative expression level. The expression level was normalized by using the control gene actin and calculating the relative expression of the genes by the 2^−ΔΔCτ^ method.

**Table 1:** Primers used in real-time quantitative PCR of melatonin biosynthesis-related genes and those involved in the regulation of development and ripening.

Gene name	Primer sequence (5′–3′)	Functional annotations
*TDC*	F: GCTCGGTTGCTGCTGGTGATG R: CATATTGTTCCTGGCTTGACACATTGG	Melatonin biosynthesis
*T5H*	F: CCGCAACCGCACAACTCCTAG R: AATACCGCTTGATCCTTGGCTTCAG	Melatonin biosynthesis
*SNAT*	F: TCCGAACCGAATGTAATGTGGCTAC R: CCTCTGGTGGAGATGTTGATGTGTATG	Melatonin biosynthesis
*ASMT*	F: GCCAGTGATTCAGGAATGATGAACTTG R: GCACTATCCTGCAAGTTAGCAACAAC	Melatonin biosynthesis
*PAL*	F: CTTCTGTGGTGCTGTTTGATG R: AGGGTGGTGCTTCAGTTTATGT	Phenols biosynthesis
*CHS*	F: CATACCCCGACTACTACTTTCGT R: CGCACATACTGGGATTCTCTT	Phenols biosynthesis
*NCED1*	F: GACACCTTTTGCTTCCACTTG R: GGATTTCGGACAACACACTCT	ABA biosynthesis
*NCED2*	F: ACTGCTTCTGCTTCCATCTCT R: AGACACTCGTCGCATTCATT	ABA biosynthesis
*NCED3*	F: CAAACCCTATCACCCTTCTCAC R: TGGGCAACTTCTGCTTCTTT	ABA biosynthesis
*NCED4*	F: GAACCGTGGCCCAAAGTTTC R: TCACGGCGTTCACAATCTGA	ABA biosynthesis
*SnRK2.6*	F: GCTACACTCGCAACCAAAATC R: ACCCCACAAGACCAGACATC	Ripening regulator
*GAMYB*	F: ATCCAATGTGAAACCTGAACCA R: CCAGCCAATCTGAACCAAGTAA	Ripening regulator
*RBOH*	F: TGGCGACGAGCATGGATAGTTT R: AGGGTTTCAGCAGCACCTTTGG	Signaling H_2_O_2_ accumulation
*Actin*	F: GCTAATCGTGAGAAGATGAC R: AGCACAATACCAGTAGTACG	Housekeeping gene

### Endogenous accumulation of phenolic compounds

Half gram (0.5 g) of strawberry powder was extracted with 1 mL methanol (HPLC grade) for 30 min in ultrasonic bath. The extract was filtered by Whatman filter (No. 4) and then filtered by 0.45 µm Millipore filter. A gradient solvent system was employed with solvent A: acetonitrile and solvent B: water-acetic acid (97.5:2.5, v/v). The total run time was 35 min. The elution program was: 10 A/90 B (0 min), 40 A/60 B (15 min), 70 A/30 B (24 min) and 10 A/90 B (35 min) with flow rate of 0.5 mL/min. The phenolic compositions were recognized by comparing their retention times with those of the standards. Standards tests were performed at a concentration of 6.25–800 ppm. HPLC analysis was carried out using Knauer liquid chromatography apparatus consisting of a 1050 Smartline Pump, a 2600 Smartline Photodiode Array Detector, DGU-14A degasser. The separation was achieved on a 150 mm × 4.6 mm with a pre-column, Thermo Hypersil-Keystone C18 reversed-phase column provided by Knauer (Berlin, Germany). The peaks were monitored at 280 nm. Injection volume was 15 μL, and the temperature was maintained at 25 °C. The EZchrom Elite software were used to collect and integrate data.

### Statistical analysis

A completely randomized design with five replications was used to conduct this study. Statistical data analysis used SAS (Version 9.4; SAS Institute, 2003) software. The data were subjected to two-way analysis of variance (ANOVA) and presented as means ± standard error. To identify the difference between the means, the least differences (LSD) were calculated at the 0.05 level.

All local, national or international guidelines and legislation were adhered to in the production of this study.

## Results

### Endogenous anthocyanin content

**Figure 1:**
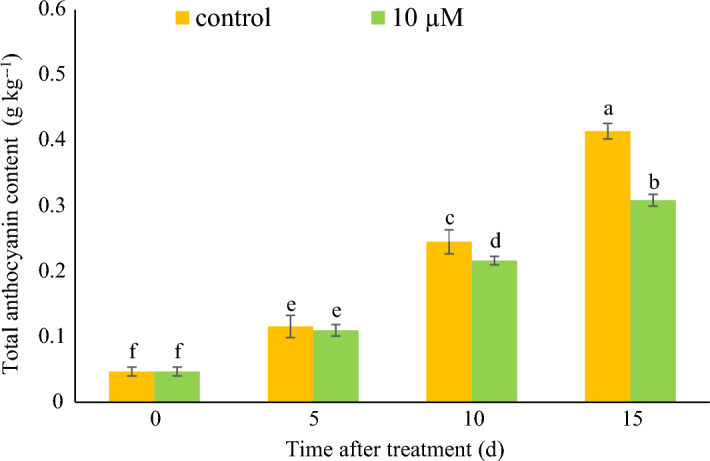
Total anthocyanin content of strawberry fruit treated with 0, 10 µM melatonin at different time after treatment. Data represent the means of five replicates and their standard errors. Vertical bars without the same letter on top indicate significant differences at *p* < 0.05.

The anthocyanin content increased during sampling in both control and melatonin treatment (Fig. 1). Anthocyanin of control and 10 μM melatonin showed no significant differences at 0 and 5 days after treatment. While, it was lower in 10 μM melatonin treatments than control at 10 and 15 days after treatment.

### Fruit firmness, TSS, TA, and color

The fruit firmness decreased during fruit ripening. The firmness of 10 µM melatonin was higher than control at 5 and 10 days after treatment (Table 2). TSS content continually increased during 15 days (Table 2). However, the TSS content was lower in 10 μM melatonin than control at 10 days (5% vs. 5.4%) and 15 days (6.4% vs. 7%). The TA content decreased in the fruit. The lower TA (0.6%) was observed in the control at 15 day after treatment, while it was 0.9% in 10 µM melatonin treated fruit at same time. The first redness signs (a* index) was observed at 10 days after treatment and a* increased thereafter. The a* in 10 µM melatonin treatment was lower than control at 10 and 15 days after treatment (Table 2).

**Table 2:** Effect of melatonin treatment on total soluble solid (TSS), titratable acidity (TA), firmness, and fruit color (a*) of strawberry fruit at different time after treatment.

Time after treatment (days)	Treatment	TSS (%)	TA (% citric acid)	Firmness (N)	a*
0	0	4.26 ± 0.05 f	2.16 ± 0.14 c	40.38 ± 2.16 a	–^a^
	10	4.24 ± 0.05 f	2.22 ± 0.16 c	40.88 ± 2.62 a	–
5	0	4.66 ± 0.09 e	3.03 ± 0.22 a	26.36 ± 1.11 c	–
	10	4.73 ± 0.08 e	2.88 ± 0.32 b	28.75 ± 1.07 b	–
10	0	5.43 ± 0.13 c	1.16 ± 0.18 e	10.33 ± 0.64 e	12.56 ± 0.87 c
	10	5.03 ± 0.13 d	1.65 ± 0.09 d	12.28 ± 0.42 d	08.84 ± 0.64 d
15	0	7.04 ± 0.16 a	0.65 ± 0.07 g	7.63 ± 0.22 g	38.66 ± 2.68 a
	10	6.43 ± 0.26 b	0.86 ± 0.08 f	8.21 ± 0.37 f	34.18 ± 3.22 b

According to LSD test, values in the same column with different letters are statistically different (*p* < 0.05).

^a^Was not measured.

### Accumulation of endogenous melatonin

**Figure 2:**
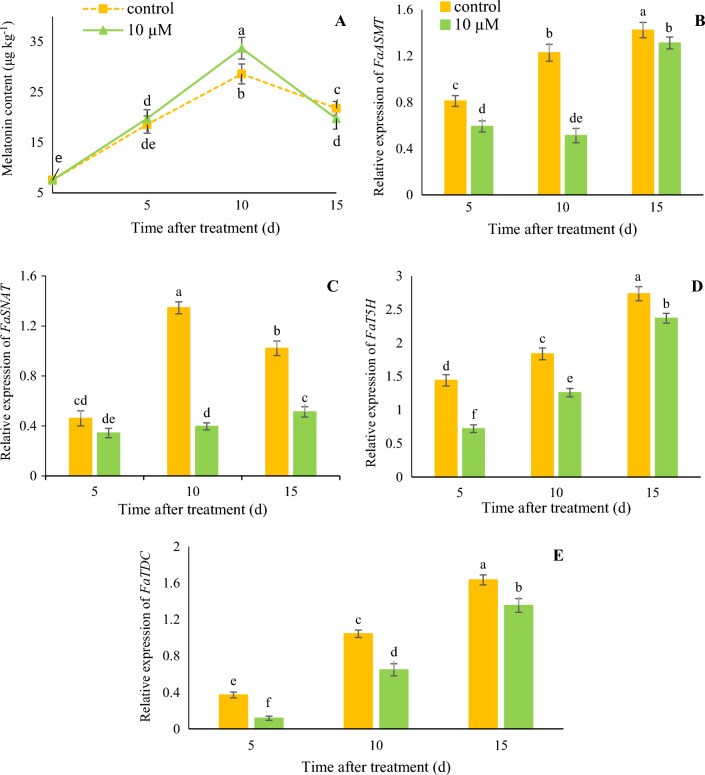
Effect of melatonin treatment on endogenous melatonin accumulation (**A**) and relative expression of *FaASMT* (**B**), *FaSNAT* (**C**), *FaT5H* (**D**), and *FaTDC* (**E**) genes of strawberry fruit at different time after treatment. Data represent the means of five replicates and their standard errors. Vertical bars without the same letter on top indicate significant differences at *p* < 0.05.

Endogenous melatonin accumulated during fruit ripening by 10 days after treatment and declined thereafter at 15 days after treatment (Fig. 2A). Endogenous melatonin in 10 µM melatonin treatment was higher than control at 10 days after treatment (33.7 μg/g FW vs. 28.6 μg/g FW).

The expression level of genes (*TDC*, *T5H*, and *ASMT*) involved in melatonin biosynthesis increased during fruit ripening while their expressions in 10 µM melatonin were lower than control at all days after treatment (Fig. 2B, D and E). The expression of *SNAT* increased during fruit ripening by 10 days after treatment but declined thereafter at 15 days after treatment (Fig. 2C).

### Expression of the *GAMYB* and *SnRK2.6* genes during fruit development

**Figure 3:**
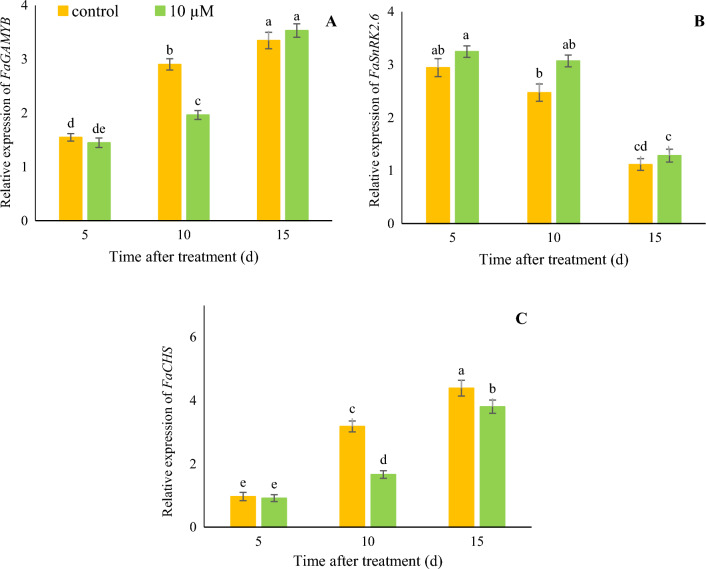
Effect of melatonin treatment on the relative expression of *FaGAMYB* (**A**), *FaSnRk2.6* (**B**) and *FaCHS* (**C**) of strawberry fruit at different time after treatment. Data represent the means of five replicates and their standard errors. Vertical bars without the same letter on top indicate significant differences at *p* < 0.05.

As shown in Fig. 3A, the expression of the *GAMYB* gene increased during the ripening process. Fruit treated with 10 μM melatonin showed 0.65-fold less expression (1.9) at 10 days compare to control fruit (2.9). Expression of the *SnRK2.6* gene (Fig. 3B) was higher at the early stages than at the ripening stages. The fruit treated with 10 μM melatonin displayed 1.25-fold higher expression of the *SnRK2.6* gene than the control samples. Our results showed that the relative expression of the *CHS* gene in 10 μM melatonin decreased by 0.52-fold and 0.72-fold than control at 10 and 15 days (Fig. 3C).

### Accumulation of H_2_O_2_ and endogenous ABA

**Figure 4:**
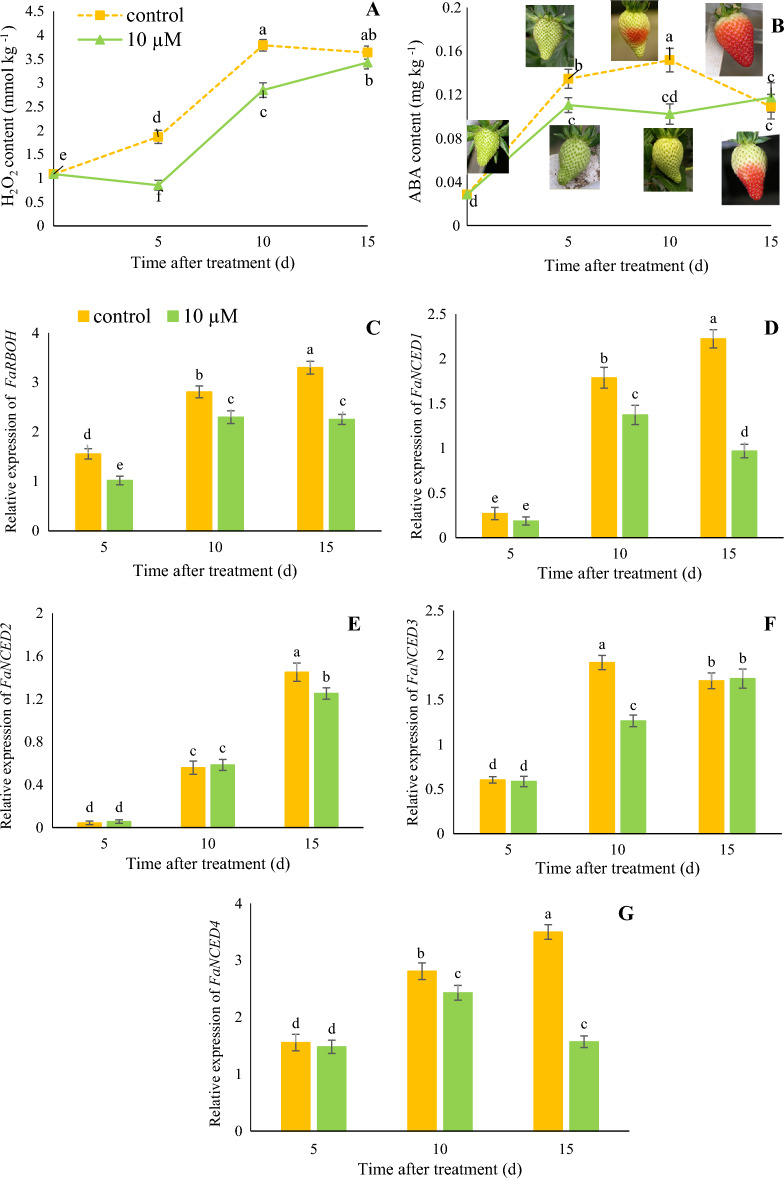
The effects of melatonin injection on H_2_O_2_ accumulation (**A**), ABA concentration (**B**), relative expression of *FaRBOH* (**C**) *FaNCED1* (**D**), *FaNCED2* (**E**), *FaNCED3* (**F**), and *FaNCED4* (**G**) gens of strawberry fruit at different time after treatment. Data represent the means of five replicates and their standard errors. Vertical bars without the same letter on top indicate significant differences at *p* < 0.05.

As shown in Fig. 4A, the endogenous accumulation of H_2_O_2_ increased during ripening. Fruit treated with 10 µM melatonin exhibited a significantly (*p* < 0.05) lower H_2_O_2_ accumulation at 5 days (0.85 mmol/kg vs. 1.86 mmol/kg) and 10 days (2.85 mmol/kg vs. 3.79 mmol/kg) compared to control samples. Also, the relative expression of the *FaRBOH* gene as a signaling H_2_O_2_ accumulation molecule in fruit treated with 10 µM melatonin decreased by 0.82-fold and 0.68-fold than control samples at 10 and 15 days (Fig. 4C). As shown in Fig. 4B, ABA content in 10 μM melatonin treatment and control increased continuously until 10 days after treatment, then gradually decreased at 15 days. Fruit treated with 10 µM melatonin exhibited a significantly (*p* < 0.05) lower ABA accumulation at 5 days (0.11 mg/kg vs. 0.14 mg/kg) and 10 days (0.10 mg/kg vs. 0.15 mg/kg) compared to control samples. The delayed ripening of strawberry fruits in response to the injection of 10 μM melatonin was accompanied by a lower endogenous content of ABA, resulting from a lower expression of *NCED1*, *2, 3*, and *4* genes (Fig. 4D–G).

### The activity of PAL, TPC and DPPH scavenging capacity

**Figure 5:**
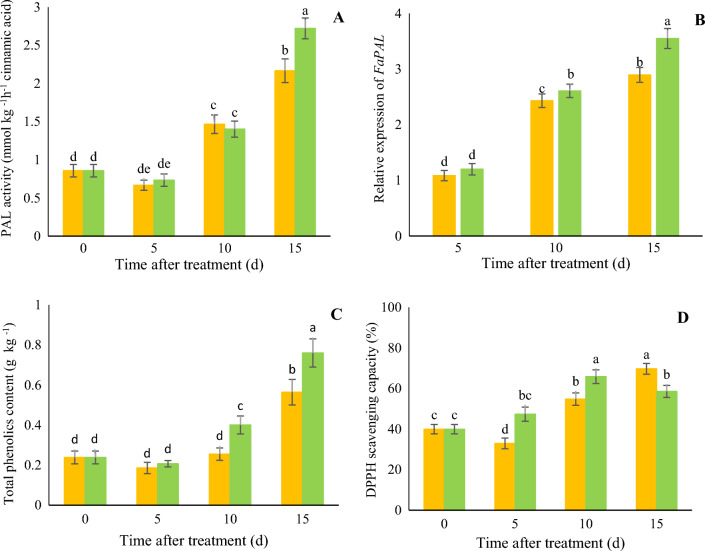
Effect of melatonin treatment on the PAL activity (**A**), *FaPAL* (**B**), total phenolics content (**C**), and DPPH scavenging capacity (**D**) of strawberry fruit at different time after treatment. Data represent the means of five replicates and their standard errors. Vertical bars without the same letter on top indicate significant differences at *p* < 0.05.

Strawberries treated with 10 µM melatonin exhibited higher PAL enzyme activity than control samples at 15 days (Fig. 5A), ascribed to 1.22-fold higher *PAL* gene expression (Fig. 5B) but there was no significant different in the PAL activity between fruit treatment and control samples at 5 and 10 days after treatment. As shown in Fig. 5C, fruit treated with 10 µM melatonin exhibited higher phenol accumulation at 10 days (40 g/kg vs. 25 g/kg) and 15 days (76 g/kg vs. 56 g/kg) during fruit development. DPPH scavenging capacity was higher in melatonin treated fruit at 5 (47% vs. 33%) and 10 days (66% vs. 55%), but at 15 day, the control samples had a higher DPPH scavenging capacity (Fig. 5D).

### Phenolic compounds

The HPLC results showed that the phenolic constituent changed during different days after treatment. The content of quercetin, gallic acid, caffeic acid, and chlorogenic acid increased during ripening, but rosmarinic acid increased until 5 days, and decreased slightly. The fruit treated with 10 µM melatonin showed higher content of quercetin than control at 10 days (0.05 g/kg vs. 0.04 g/kg) and 15 days (0.07 g/kg vs. 0.05 g/kg) (Table 3). The higher concentration of gallic acid was observed in melatonin treatment than control samples (0.29 g/kg vs. 0.24 g/kg) at 15 days after treatment. The total rosmarinic acid and cafeic acid concentrations in 10 µM melatonin treatment were higher than control at 5 and 10 days while no significant difference was observed at 15 days. There was no significant differences in chlorogenic acid of melatonin treatment and control at 5 and 10 days while it was higher in melatonin treatment than control at 15 days (0.13 g/kg vs. 0.10 g/kg) (Table 3).

**Table 3:** Concentrations (g kg^−1^) of selected phenolic compounds of strawberry fruit at different time after treatment.

Time after treatment (days)	Melatonin (µM)	Quercetin	Gallic acid	Rosmarinic acid	Caffeic acid	Chlorogenic acid
0	0	0.014 ± 0.001 e	0.135 ± 0.01 c	0.039 ± 0.006 d	0.043 ± 0.003 f	0.009 ± 0.0001 f
	10	0.014 ± 0.001 e	0.136 ± 0.01 c	0.039 ± 0.006 d	0.043 ± 0.003 f	0.009 ± 0.0001 f
5	0	0.029 ± 0.003 d	0.103 ± 0.01 d	0.113 ± 0.024 b	0.072 ± 0.001 e	0.018 ± 0.003 e
	10	0.032 ± 0.004 d	0.140 ± 0.01 c	0.137 ± 0.023 a	0.092 ± 0.001 d	0.018 ± 0.004 e
10	0	0.036 ± 0.003 c	0.233 ± 0.02 b	0.083 ± 0.009 c	0.158 ± 0.002 b	0.062 ± 0.001 d
	10	0.051 ± 0.004 b	0.156 ± 0.01 c	0.103 ± 0.012 b	0.131 ± 0.002 c	0.081 ± 0.001 c
15	0	0.051 ± 0.006 b	0.243 ± 0.02 b	0.076 ± 0.008 c	0.210 ± 0.002 a	0.104 ± 0.002 b
	10	0.066 ± 0.006 a	0.289 ± 0.02 a	0.082 ± 0.006 c	0.197 ± 0.002 a	0.132 ± 0.002 a

The results are from HPLC analyses of two extracts of each sample. According to LSD test, values in the same column followed by different letters are statistically different (p < 0.05).

## Discussion

In recent years, melatonin as a signal and antioxidant molecule has been shown to play a role in the ripening of fruit^26–28^. Fruit treated with 10 μM melatonin showed lower anthocyanin content than control. There are various reports on melatonin’s different roles in regulating crops’ ripening and its effect on the reduction of anthocyanins in the fruit ripening process^29^. The decrease of anthocyanin can be caused by the increase of cytokines due to exogenous melatonin, because there is a positive correlation between melatonin and cytokinin^30^. Consistent with our results, Tijero et al.^5^ reported that treatments with melatonin (10 and 100 μM) reduced anthocyanin content and delayed ripening of cherry fruit due to an increase in the endogenous cytokinin. This findings were against previous report that 1000 μM melatonin enhanced anthocyanin content in strawberry^4^.

Since tissue softness is one of the most important ripening indicators, the samples treated with 10 µM melatonin had less softness than the control. Our results showed that samples treated with 10 μM melatonin had lower TSS content and color (a*) than the control. Sucrose, fructose and glucose are the main soluble sugars in strawberries^31^. Melatonin can delay ripening by reducing respiration and ethylene production, thus delaying fruit softening and color change and causing changes in TSS and TA content^32^. Zhai et al.^33^ reported that the use of melatonin (100 μM) in pear fruit maintained the firmness of the treated fruits, and this effect of melatonin was through reducing the expression of poly-galactronase and cellulase genes, which are effective in reducing the firmness of the fruit. Melatonin is an amphiphilic molecule, which allows it to enter almost all intracellular compartments by crossing membranes^34^. Zhou et al.^35^ reported that melatonin acts as a mediator and is able to inhibit the reduction process of organic acids in fruit and increase the storage life. In addition, the effect of melatonin on delay ripening has been reported in other fruits such as mango^32^, and sweet cherry^5^.

The results demonstrated that application of melatonin (10 μM) markedly decreased H_2_O_2_ accumulation. Melatonin has direct antioxidant activity^7^ and can decrease the accumulation of OH, O^-1^, H_2_O_2_, and NO free radicals and regulate antioxidant enzymes^36–38^. Decreasing the endogenous level of H_2_O_2_ can reduce anthocyanin, and plays a vital role in preventing tomato fruit ripening^39^. Fruit ripening induces an oxidative condition and may accompanied by an increase in H_2_O_2_^6^_._ We found that 10 μM melatonin treatment delayed the ripening by suppressing H_2_O_2_ signaling, and decreasing in anthocyanin compared to the control. Similar findings have been reported about the effectiveness of melatonin in reducing H_2_O_2_ content in strawberries ^40^.

Our findings showed that treatment with 10 μM melatonin reduced the endogenous accumulation of ABA in strawberry fruit compared to control samples. Transmission and signaling of ABA is a model for non-climacteric fruit ripening such as strawberries^18^. The ripening of non-climacteric fruits has been classically considered to be mainly regulated by ABA, which is involved in the accumulation of anthocyanins and sugars^5^. Moreover, ABA is associated with fruit softening and sugar accumulation in strawberry fruit^3^. The most effect of ABA on fruit ripening is the positive regulation of anthocyanin accumulation in grapes^6^ and strawberries^18^. Thus, ABA as a ripening promoter in non-climacteric fruit involved in sugar accumulation and changing the fruit’s color by regulating the biosynthesis of anthocyanin and phenylpropanoid pathway^3^. Sandhu et al.^41^ found that ABA treatment stimulates anthocyanins and total phenolic production, enhancing the antioxidant capacities in ‘Muscadine’ grapes. Our previous results showed that a higher endogenous accumulation of ABA and a higher accumulation of anthocyanin (due to higher expressions of *PAL* and *CHS* genes) attributed to a higher expression of the *GAMYB* gene and a lower expression of *SnRK2.6* gene, resulting from the endogenous accumulation of melatonin, involved in the ripening of strawberry fruits^4^. Based on our results, strawberry fruit ripening is dependent on melatonin concentration. Similar to our results, another study showed that different concentrations of methyl jasmonate (MeJA) had different effects on grape fruit ripening. García-Pastor et al.^13^ showed that MeJA at 5 and 10 mM delayed berry ripening and decreased berry weight and volume as well as vine yield, however, treatments with MeJA at 1, 0.1 and 0.01 mM accelerated ripening and increased total phenolics and individual anthocyanin concentrations. Regarding the mechanisms of action of melatonin during fruit ripening and its relationship with ABA, Fu et al.^42^ reported that ABA may act as a downstream signal of melatonin in response to stress. Consistent with our results, Liu et al.^12^ reported that pear fruit treatment with melatonin delayed fruit ripening by reducing the endogenous accumulation of ABA and inhibiting ethylene synthesis.

According to our results, the effectiveness of melatonin in decreasing *CHS* and *SnRK2*.*6* gene expression might be the reasons for the decrease of anthocyanins during ripening. According to the literature, increasing the expression of *CHS* and *CHI* genes in fruit is effective in their coloring^43^. Zhang et al.^8^ reported that the accumulation of anthocyanins in cabbages in response to melatonin treatment was due to higher phenylpropanoid biosynthesis-related genes (*PAL, C4H, CHS, CHI*, and *F3H*). *SnRK2.6* as a negative regulator of fruit ripening, rapidly decreased during strawberry fruit development, and silencing the expression level of *FaSnRK2.6* inhibited the ripening of this fruit ^2^. Han et al. (2015)^2^ found that ABA affects the expression of *SnRK2.6*, and the expression of this gene decreases ABA concentration, fruit color and aroma metabolism genes such as *CHS* and *CHI*, which generally slow down fruit ripening. Thus, inhibition of *GAMYB* gene expression and increased *SnRK2.6* gene expression could be why 10 μM exogenous melatonin reduced endogenous ABA accumulation and delayed ripening stages of strawberry fruit. The higher *GAMYB* gene expression in the white stage of strawberry fruit may act as an essential regulator of ripening by increasing biosynthesis and ABA signaling, and activate the phenylpropanoid pathway and pigment accumulation in the fruit^44^.

In fruit treated with 10 μM melatonin, PAL enzyme activity and the TPC increased by increasing *PAL* gene expression during the ripening process. Besides, strawberries treated with 10 µM melatonin exhibited higher DPPH scavenging capacity. It has been reported that the free radical scavenging capacity is enhanced by melatonin, which makes it very effective in protecting organisms against oxidative stress, even at low concentrations^38,45^. Therefore, triggering the endogenous accumulation of melatonin in strawberries treated with 10 µM melatonin might be responsible for promoting the activity of the phenylpropanoid pathway, represented by higher PAL, and increased DPPH scavenging capacity. Consistent with our results, Sharafi et al.^46^ reported that melatonin treatment in tomato fruit increased PAL enzyme activity, phenol accumulation, and increased ATP and carbon skeleton in the fruit. Moreover, Aghdam et al.^47^ reported that applying 100 μM melatonin on pomegranate fruit increased phenylpropanoid pathway activity, PAL enzyme activity, phenol content and DPPH antioxidant capacity.

According to our results, the phenolic constituent in strawberry fruit treated with 10 µM melatonin increased. Similar to our findings, melatonin treatment in grapes increased the accumulation of chlorogenic, gallic, caffeic, cinnamic, and coumaric acid phenols and flavonoids, catechin, epicatechin, and pelargonidin by increasing the expression of *CHS* and *PAL* genes^48^. In addition, the effectiveness of melatonin in increasing phenolic compounds in plums has also been reported^49^.

## Conclusion

**Figure 6:**
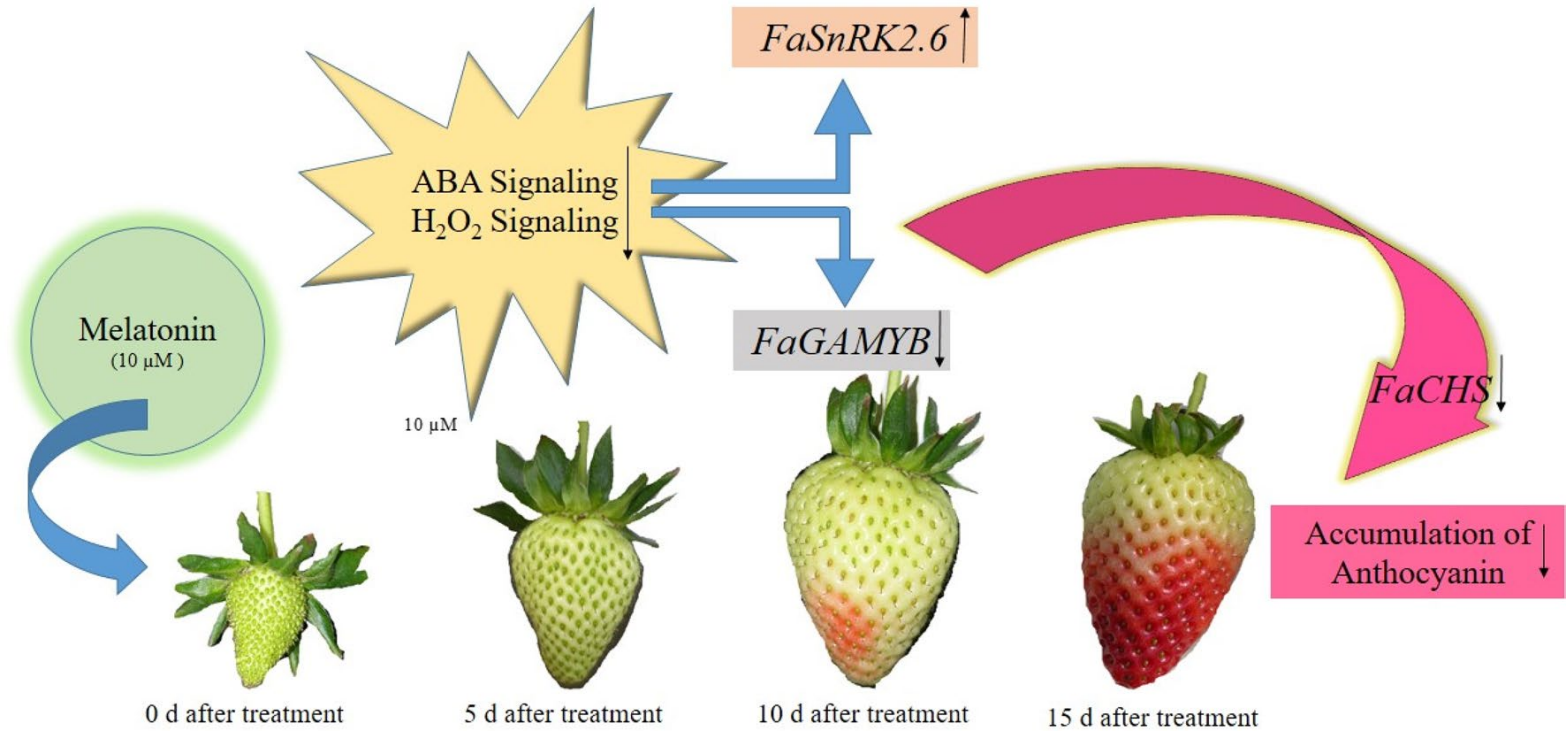
Schematic diagram for the effects of melatonin on some physiological and biochemical aspects of fruit ripening in strawberry. Melatonin treatment delayed the ripening by suppressing H_2_O_2_ and ABA signaling attributed to a higher expression of *GAMYB* gene and a lower expression of *SnRK2.6* gene caused decreased in anthocyanin.

Our results showed that the fruits treated with 10 μM melatonin had less TSS, red color and more firmness and TA than the control samples at ripening stage, so were more unripe based on ripening indicators. Melatonin could regulate the expression of some genes involved in fruit ripening, such as *CHS*, *GAMYB*, *SnRK2.6*, and *PAL* through the decrease of endogenous ABA. Furthermore, melatonin as an antioxidant, plays an essential role in accumulating antioxidants and phenolic compounds during fruit development by reducing the oxidative stress of endogenous H_2_O_2_ (Fig. 6). This article examined the role of melatonin on ABA and H_2_O_2_ in regulating the ripening process through the phenylpropanoid pathway, such as fruit coloring, and highlighting a deeper understanding of the factors that control non-climacteric fruit ripening. Thus, it can provide a tool to delay strawberry fruit ripening. As melatonin is a non-harmful compound, it will help producers and growers to adjust the harvest time based on market needs.

## Data Availability

The datasets analyzed during the current study are available from the corresponding author on reasonable request.

## Abbreviations

ABA: Abscisic acid
ASMT: *N*-Acetylserotonin methyltransferase
CHS: Chalkone synthase
NCED: 9-Cisepoxycarotenoid dioxygenase
PAL: Phenylalanine ammonia-lyase
RBOH: NADPH oxidase-dependent H_2_O_2_ production
ROS: Reactive oxygen species
TPC: Total phenol content
SNAT: Serotonin *N*-acetyltransferase
TDC: Tryptophan decarboxylase
T5H: Tryptophan 5-hydroxylase
SnRK2.6: Sucrose nonfermenting1-related protein kinase2 gene
